# Supplementary material for: Genome-Wide Identification, Evolution, and miRNA-22 Regulation of Kruppel-Like Factor (KLF) Gene Family in Chicken (Gallus gallus)
Source: Animals (Basel). 2024 Sep 6;14(17):2594. doi: 10.3390/ani14172594 (PMC11394431; doi:10.3390/ani14172594)
Supplement: Supplementary file 1 [file animals-14-02594-s001.zip › animals-3113359-supplementary.pdf]

Supplementary Material

Supplementary Table S1: The corresponding chromosome, Location and transcript of chicken KLF gene.

Chr	Location	gene	RNA_gene
1	NC_052532.1	*KLF5*	XM_417013.7
1	NC_052532.1	*KLF12*	XM_003640556.6<br>XM_040706699.2<br>XM_046902092.1<br>XM_025146779.3<br>XM_040706701.2<br>XM_015277008.4<br>XM_040706704.2<br>XM_015277019.4<br>XM_040706708.2
2	NC_052533.1	*KLF6*	XM_046921718.1<br>NM_001030969.2
2	NC_052533.1	*KLF10*	XM_427148.8<br>XM_015282990.4<br>XM_004939982.5<br>XM_015282991.4
3	NC_052534.1	*KLF11*	NM_001006417.2<br>XM_015276039.4<br>XM_015276040.4
4	NC_052535.1	*KLF3*	XM_015285673.4<br>XM_046940926.1<br>XM_040699828.2<br>XM_046940922.1<br>XM_046940925.1<br>XM_040699829.2<br>XM_046940924.1<br>XM_015285674.4<br>XM_046940923.1<br>XM_025149970.3
4	NC_052535.1	*KLF8*	XM_015278568.4<br>XM_015278569.4<br>XM_015278567.4

7	NC_052538.1	*KLF7*	XR_212609.5<br>XR_212610.5<br>XR_001467464.4<br>XR_005861270.2<br>XR_001467465.4<br>XM_015289510.4<br>XM_004942644.5<br>NM_001397491.1
8	NC_052539.1	*KLF1*	XM_046898236.1
10	NC_052541.1	*KLF13*	XM_425065.8
12	NC_052543.1	*KLF15*	XM_046900057.1<br>XM_046900050.1<br>XM_025154676.3<br>XM_046900038.1<br>XM_046900043.1<br>XM_046900047.1<br>XM_046900046.1<br>XM_046900056.1<br>XM_046900044.1<br>XM_046900042.1<br>XM_046900055.1<br>XM_046900059.1<br>XM_046900048.1<br>XM_046900045.1<br>XM_046900053.1<br>XM_046900051.1<br>XM_046900049.1<br>XM_046900040.1<br>XM_046900039.1<br>XM_046900041.1<br>XM_046900058.1<br>XM_046900052.1<br>XM_046900054.1<br>XM_004944605.4<br>XM_046900060.1
28	NC_052559.1	*KLF2*	NM_001318423.2
Z	NC_052572.1	*KLF4*	XM_004949369.5<br>XM_046905543.1
Z	NC_052572.1	*KLF9*	XM_003643019.6

Supplementary Figure S1 (A-M): Protein tertiary structure prediction of KLF1-KLF8, KLF10-KLF13 and KLF15, the most suitable template for each gene protein.

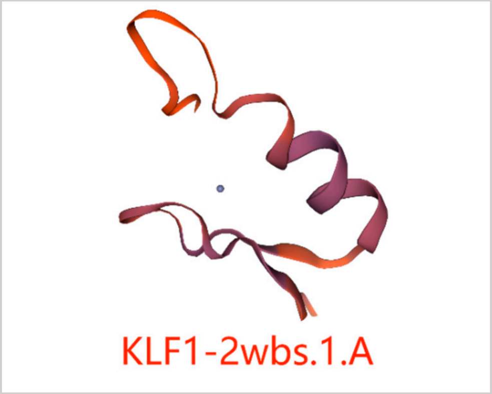

A

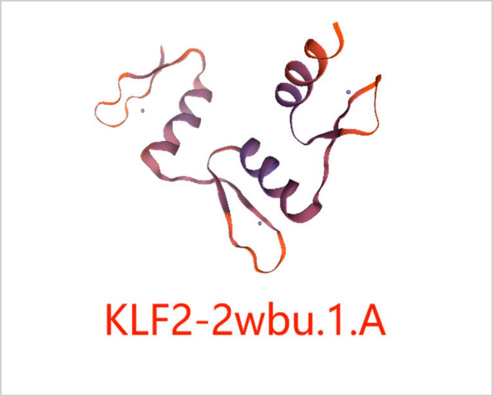

B

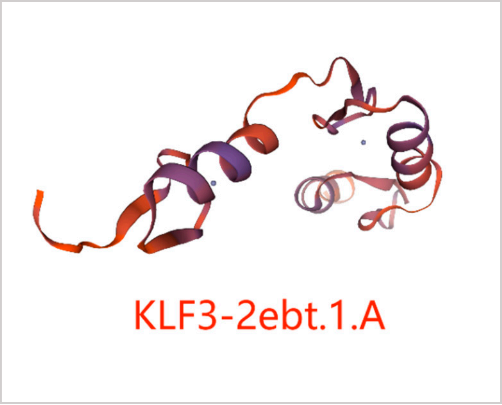

C

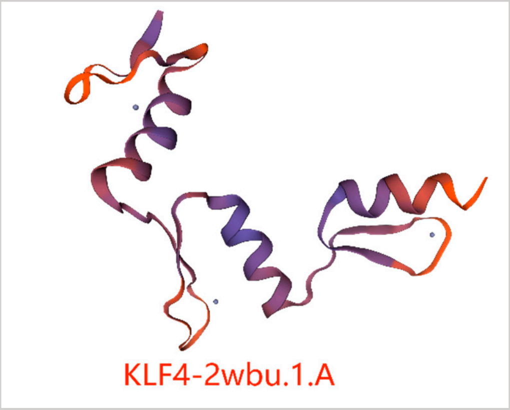

D

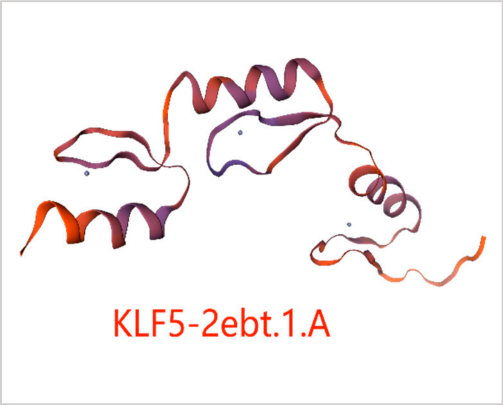

E

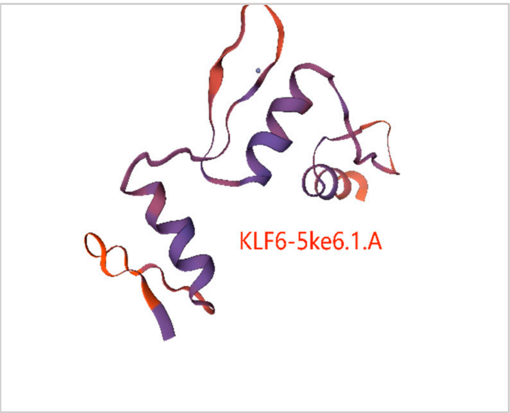

F

G
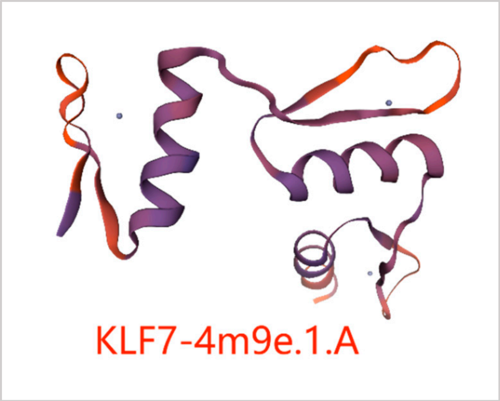
KLF7-4m9e.1.A

H
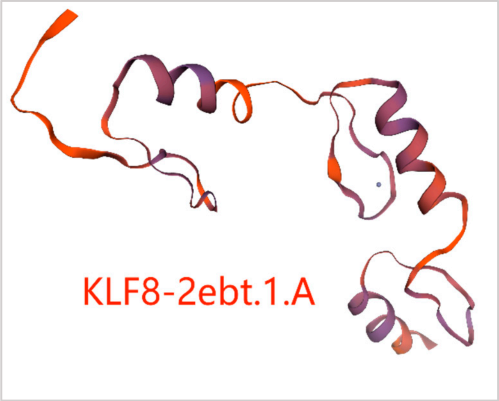
KLF8-2ebt.1.A

I
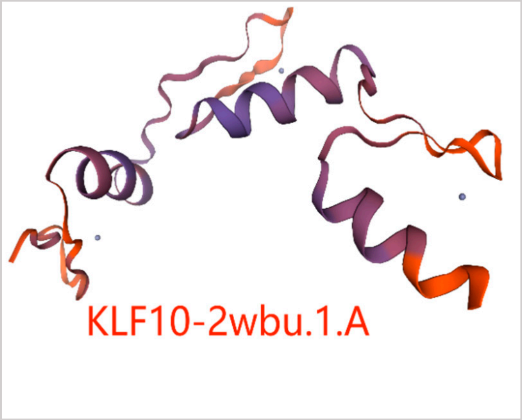
KLF10-2wbu.1.A

J
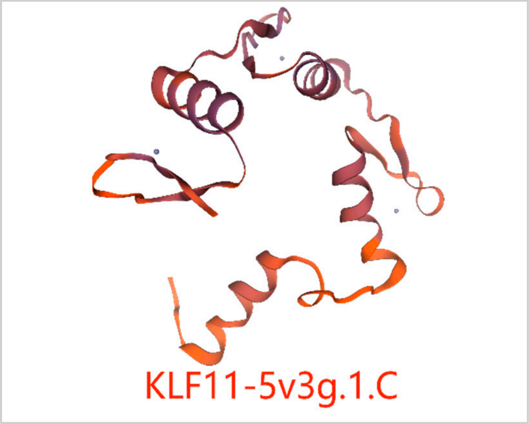
KLF11-5v3g.1.C

K
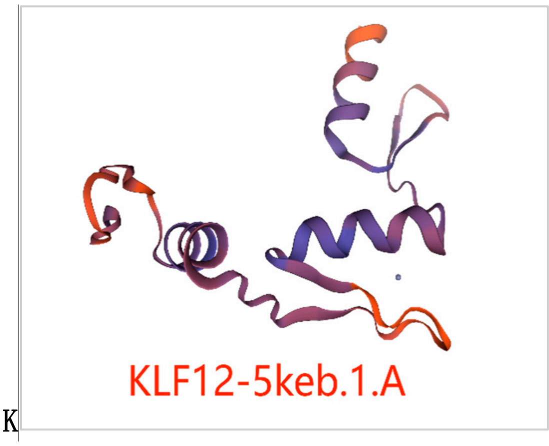
KLF12-5keb.1.A

L
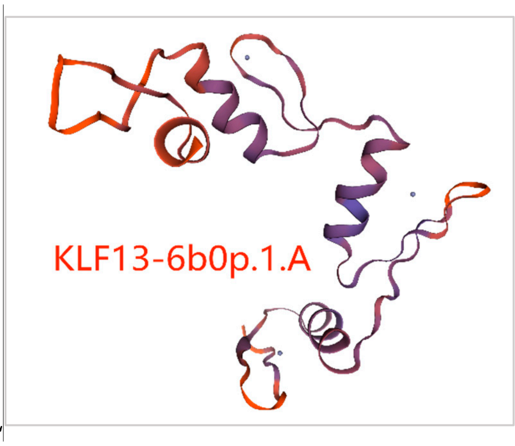
KLF13-6b0p.1.A

M
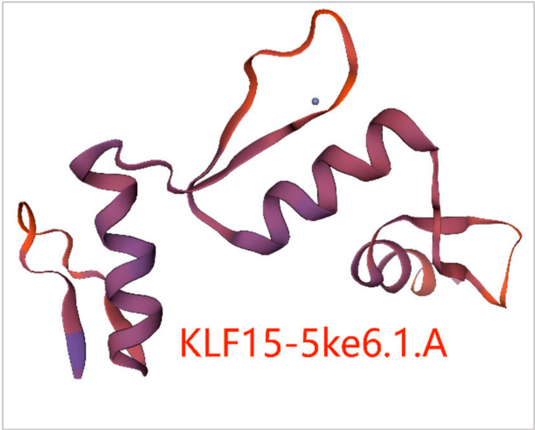
KLF15-5ke6.1.A

Supplementary Figure S2 (A-M): Prediction of the transmembrane structure of KLF1-KLF8, KLF10-KLF13 and KLF15 proteins.

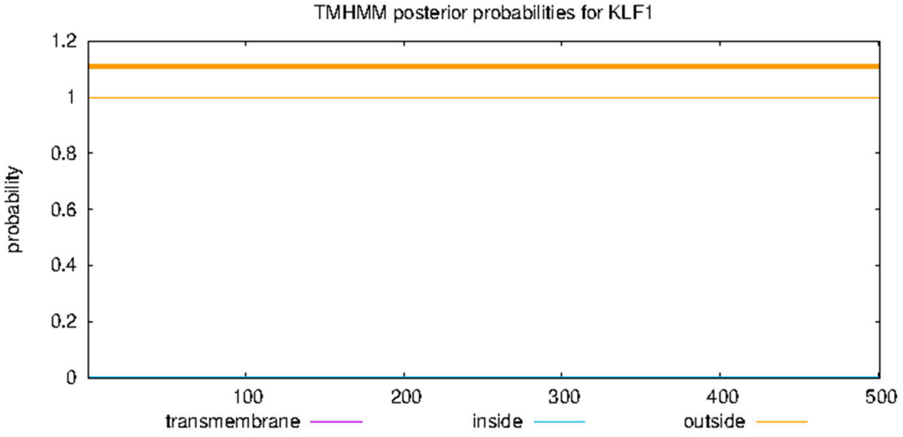

A

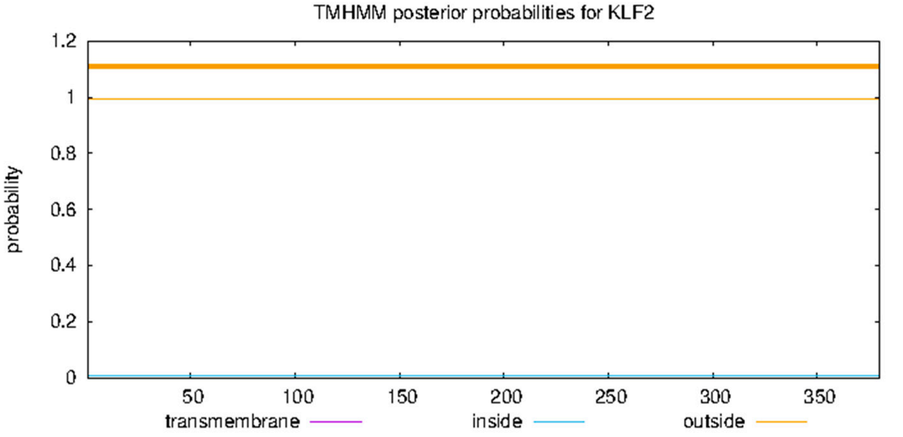

B

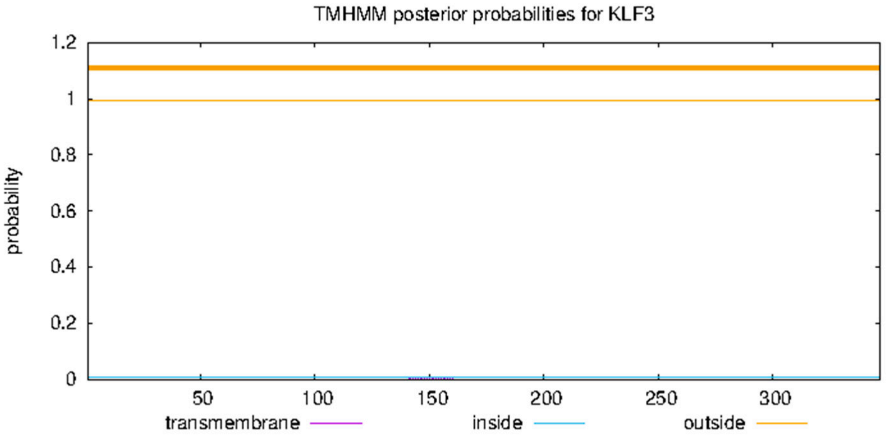
TMHMM posterior probabilities for KLF3
probability
1.2
1
0.8
0.6
0.4
0.2
0
50
100
150
200
250
300
transmembrane
inside
outside

C

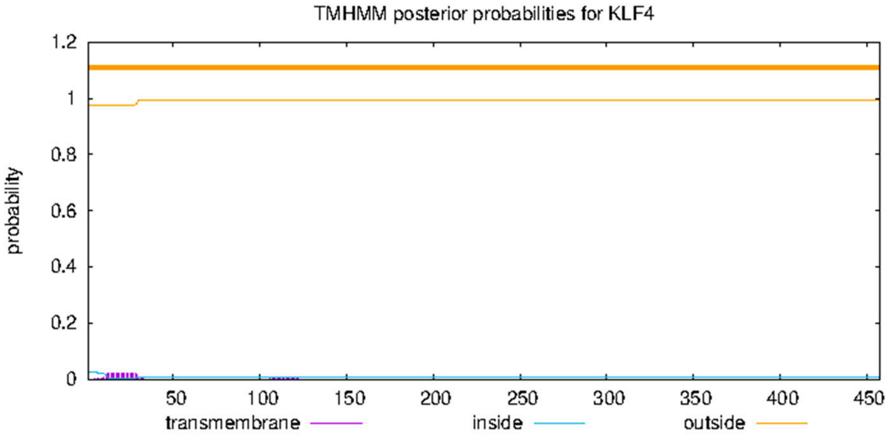
TMHMM posterior probabilities for KLF4
probability
1.2
1
0.8
0.6
0.4
0.2
0
50
100
150
200
250
300
350
400
450
transmembrane
inside
outside

D

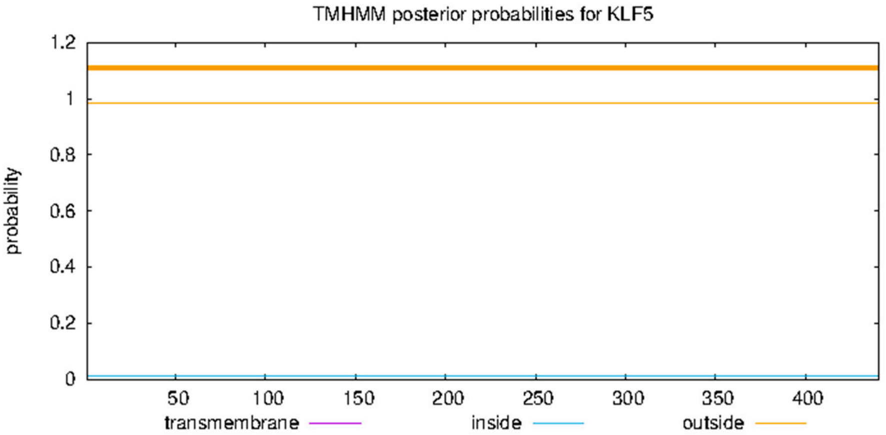
TMHMM posterior probabilities for KLF5
probability
1.2
1
0.8
0.6
0.4
0.2
0
50
100
150
200
250
300
350
400
transmembrane
inside
outside

E

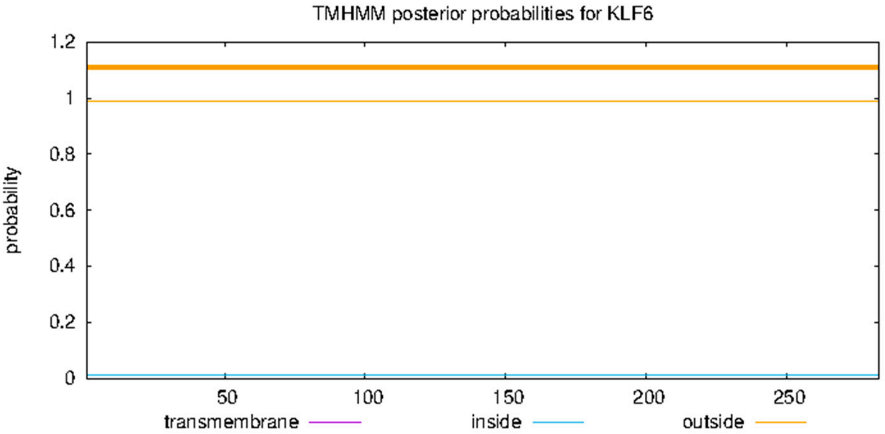
TMHMM posterior probabilities for KLF6
probability
1.2
1
0.8
0.6
0.4
0.2
0
50
100
150
200
250
transmembrane
inside
outside

F

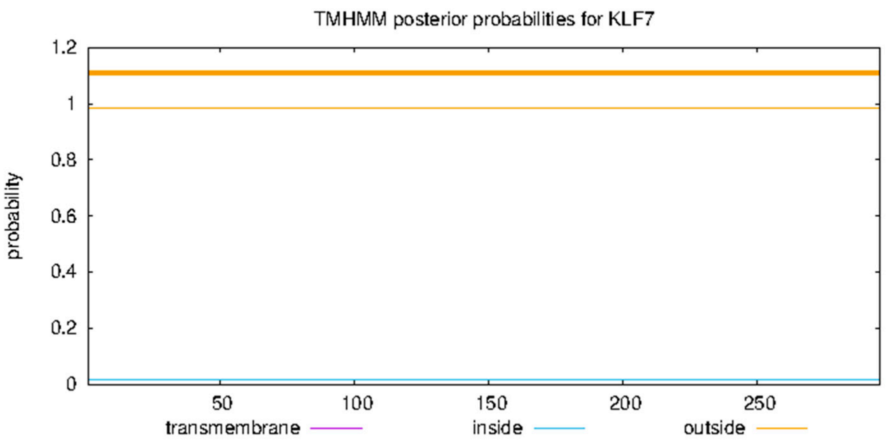
TMHMM posterior probabilities for KLF7
probability
1.2
1
0.8
0.6
0.4
0.2
0
50
100
150
200
250
transmembrane
inside
outside

G

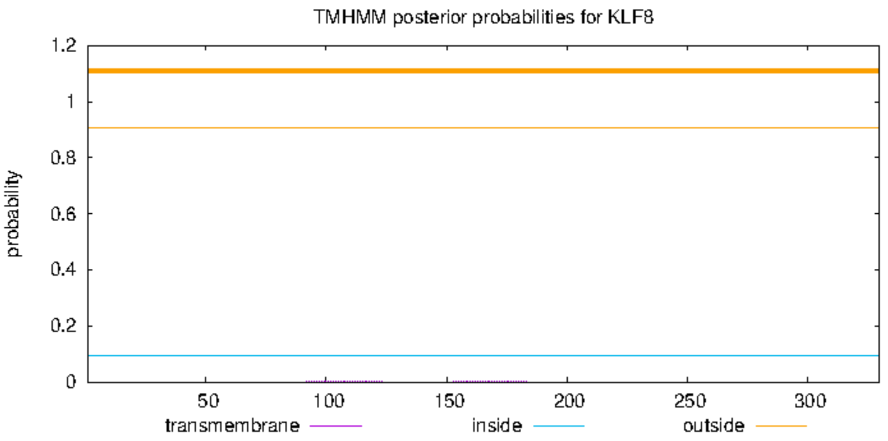
TMHMM posterior probabilities for KLF8
probability
1.2
1
0.8
0.6
0.4
0.2
0
50
100
150
200
250
300
transmembrane
inside
outside

H

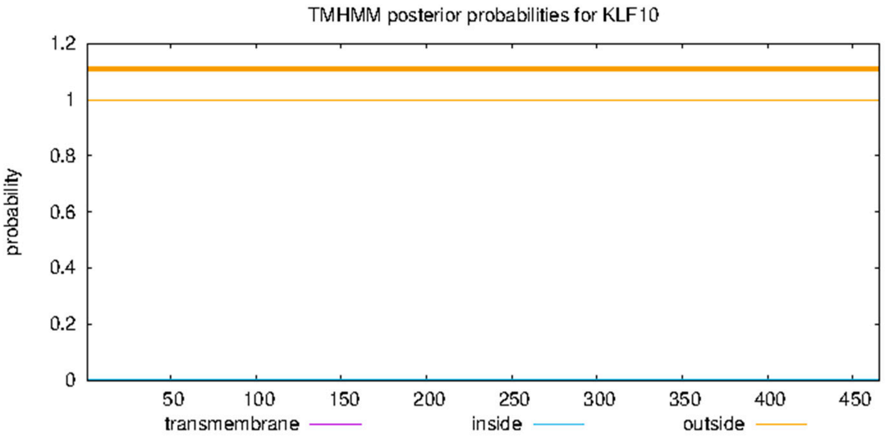
TMHMM posterior probabilities for KLF10
probability
1.2
1
0.8
0.6
0.4
0.2
0
50
100
150
200
250
300
350
400
450
transmembrane
inside
outside

I

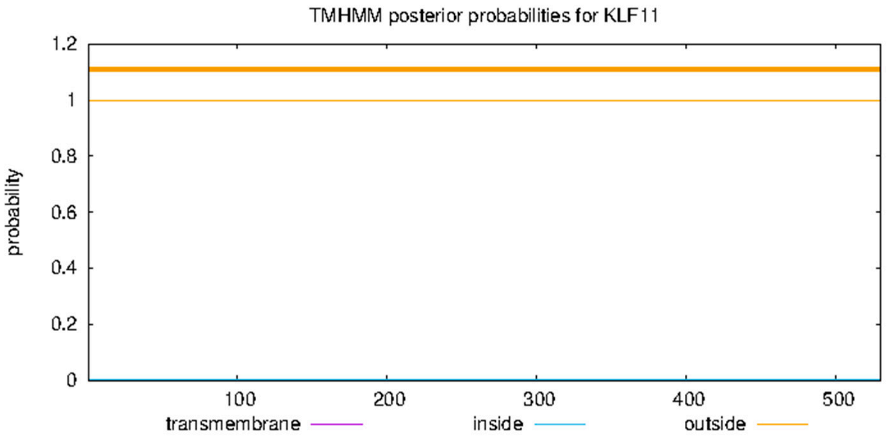
TMHMM posterior probabilities for KLF11
probability
1.2
1
0.8
0.6
0.4
0.2
0
100
200
300
400
500
transmembrane
inside
outside

J

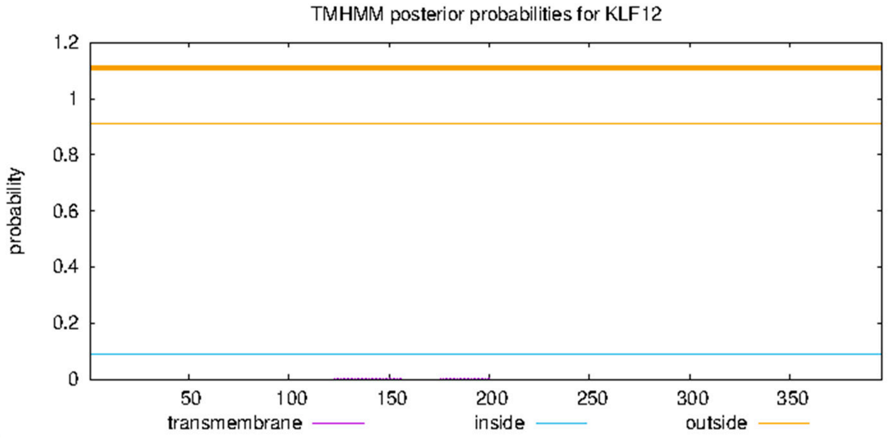
TMHMM posterior probabilities for KLF12
probability
1.2
1
0.8
0.6
0.4
0.2
0
50
100
150
200
250
300
350
transmembrane
inside
outside

K

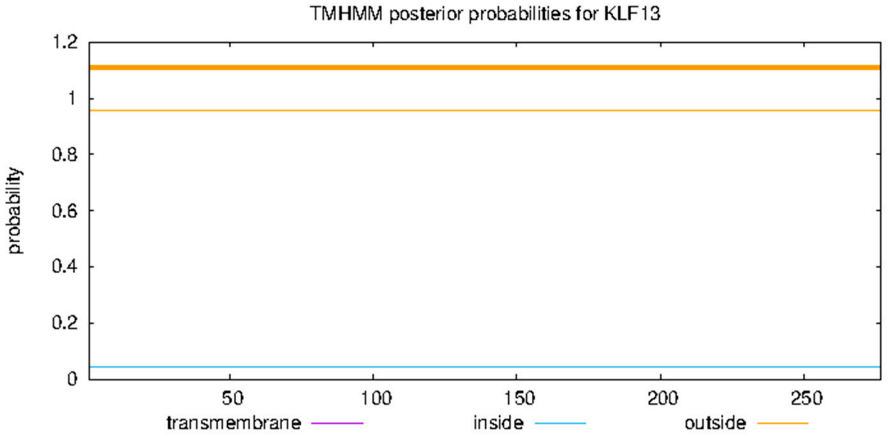
TMHMM posterior probabilities for KLF13
probability
1.2
1
0.8
0.6
0.4
0.2
0
50
100
150
200
250
transmembrane
inside
outside

L

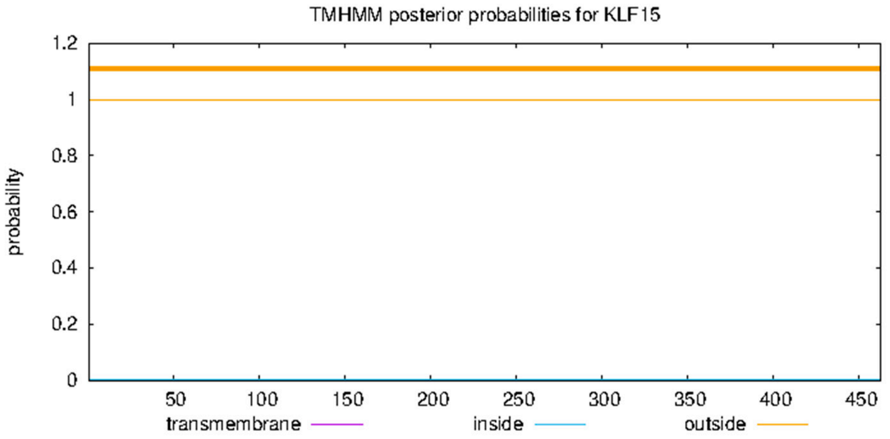
TMHMM posterior probabilities for KLF15
probability
1.2
1
0.8
0.6
0.4
0.2
0
50
100
150
200
250
300
350
400
450
transmembrane
inside
outside

M
